# Seasonal variation of heavy metals and metallothionein contents in Asian swamp eels, *Monopterus albus* (Zuiew, 1793) from Tumpat, Kelantan, Malaysia

**DOI:** 10.1186/s40360-019-0286-x

**Published:** 2019-01-29

**Authors:** Ai Yin Sow, Ahmad Ismail, Syaizwan Zahmir Zulkifli, Mohammad Noor Amal, Kamarul Hambali

**Affiliations:** 1 Faculty of Agro-Based Industry, Jeli Campus, 17600 Jeli, Kelantan Malaysia; 2 Faculty of Science, Universiti Putra Malaysia, 43400 Serdang, Selangor Malaysia; 3 Fakulti of Earth Science, Universiti Malaysia Kelantan, Locked Bag No.100, Jeli Campus, 17600 Jeli, Kelantan Malaysia

**Keywords:** Heavy metals, Metallothionein, Asian swamp eel, Paddy seasons, Kelantan

## Abstract

**Background:**

Levels of toxic metal exposure in indigenous inhabitants are key bioindicators of the severity of environmental contamination. This study measured the seasonal variation of heavy metals and metallothionein (MT) contents in Asian swamp eels (*Monopterus albus*) from a paddy field situated in Tumpat, Kelantan, Malaysia, to identify prevalence, patterns and associations and togain insight on the suitability of MT as a biomarker for metal exposure.

**Methods:**

Gill, muscle and liver tissues of *M. albus* (*n* = 50) sampled during the ploughing, seedling, growing and harvesting phases of rice growing were collected. The concentrations of copper (Cu), zinc (Zn), lead (Pb), nickel (Ni), and cadmium (Cd) in these tissues were determined by flame atomic absorption spectrometry. MT from each sample was isolated and purified, and subsequently quantitated using UV spectrophotometry. Associations between metal and MT concentrations, season and tissue type were evaluated using Pearson correlation and ANOVA with post-hoc Tukey HSD analysis.

**Results:**

Zn was present in higher quantities in gill and liver tissues, while Cu levels were elevated solely in liver. Patterns of non-essential metal accumulation were varied: Cd was detected in low concentrations in all tissues, while Pb and Ni were abundant in gill tissues across all seasons. MT concentration in liver tissue was consistently higher than that found in muscle or gill tissue, except during the growing phase. Moreover, significant correlations (*P* < 0.05) were observed for Cd, Ni, and Zn when MT was employed as metal exposure biomarker. However, no significant association was found between high Pb and Ni levels and MT concentration in gill tissue.Variation of bioaccumulation rates of heavy metals among the different tissues was observed. Some of these metal concentration differences were found to be associated with MT concentration and, by extension, to its high metal-binding capacity.

**Conclusions:**

Significant liver MT-Zn, MT-Cd, and MT-Ni correlations found in this study emphasised the role of metallothionein as a biomarker for exposure of zinc, cadmium and nickel metals in *M. albus*.

## Background

*Monopterus albus*, which is also known as Asian swamp eel, refers to a kind of fish that lives in freshwater, particularly in paddy fields. Paddy cultivation consists of several stages, namely, tilling the flooded soils or puddling, transplanting rice, and harvesting when the time is right [1, 2]. As for the Asian swamp eels that dwell in paddy fields, they have been exposed to various pollutants due to vast agrochemical usage of pesticides, fertilisers, herbicides, and polluted water.

The repetitive use of agrochemicals for paddy cultivation has escalated the amounts of pollutants in its soils. For instance, ploughing soils can cause pollutants from prior cycles of paddy to resurface, while dependency on chemical fertilisers for seedling and growing seasons adds to the amounts of pollutants. Fish uptakes important micronutrients, such as Zn, Cu, Pb, Cd, and Hg, for metabolism functions from its diet or surrounding (sediments and water), which will eventually accumulate in tissues [3–5]. This suggests that high accumulation of metals in tissues may turn into harmful toxic, for example, the high levels of Cu and Zn discovered by Pipe et al. [6] in fish tissues.

Metals accumulate in fish through several pathways, including exchange of metal ions via skin and gills, food consumption, and suspended particulate matter [7]. Additionally, Çoğun et al. [8] claimed that several factors dictate the bioaccumulation of metals, for instance, bioavailability of metals, as well as biotic and abiotic aspects including age, size, and feeding habits of species, and water temperature. Besides, liver has been reported to accumulate the highest levels of metals and thus, commonly examined to investigate bioaccumulation processes [9]. Miller et al. [10] asserted that liver is an exceptional indictor to determine inactivation and storage of metals accumulated.

Similar to other species of anguillid eel (*A. marmorata*), the Asian swamp eel has higher life longevity. At night, the fish migrate from one habitat to another for search of food, thus higher chances to get exposed to harmful pollutants. Hogstrand and Haux [11] reported that the levels of metallothionein (MT) or those similar to MT may increase in tissues due to excessive accumulation of heavy metals, such as Zn, Cu, Cd, and Hg.

According to Ureña et al. [12], MT has exhibited exceptional biochemical response towards exposure of metals. Some features of MT are listed as follows: low protein molecular weight that ranges from 6000 to 7000, rich in cysteine, resistant to heat, no disulfide bonds or aromatic amino acids, and selectively binds with heavy metals [13, 14]. In addition, MT stores and supplies important metals (Zn and Cu) for synthesis of protein, metabolism of nucleic acid [15], as well as metals detoxification. Although the analyses of MT in the species of Anguilla (eels) have begun receiving vast attention, such as that investigated by Langston et al. [16] and Ureña et al. [12]; the functions of MT in *M. albus* organs seem to be unpopular in the research arena*.*

As such, this study has two objectives, which are: 1) to examine the content of metals (Zn, Cu, Cd, Ni, and Pb) in muscle, gill, and liver of *M. albus* obtained from a paddy field located at Tumpat, Kelantan during four varied seasons, and 2) to determine the presence of MT and some other metal-binding proteins in muscle, liver, and gills of *M.albus.*

## Materials and methods

### Study area and sampling location

**Fig. 1:**
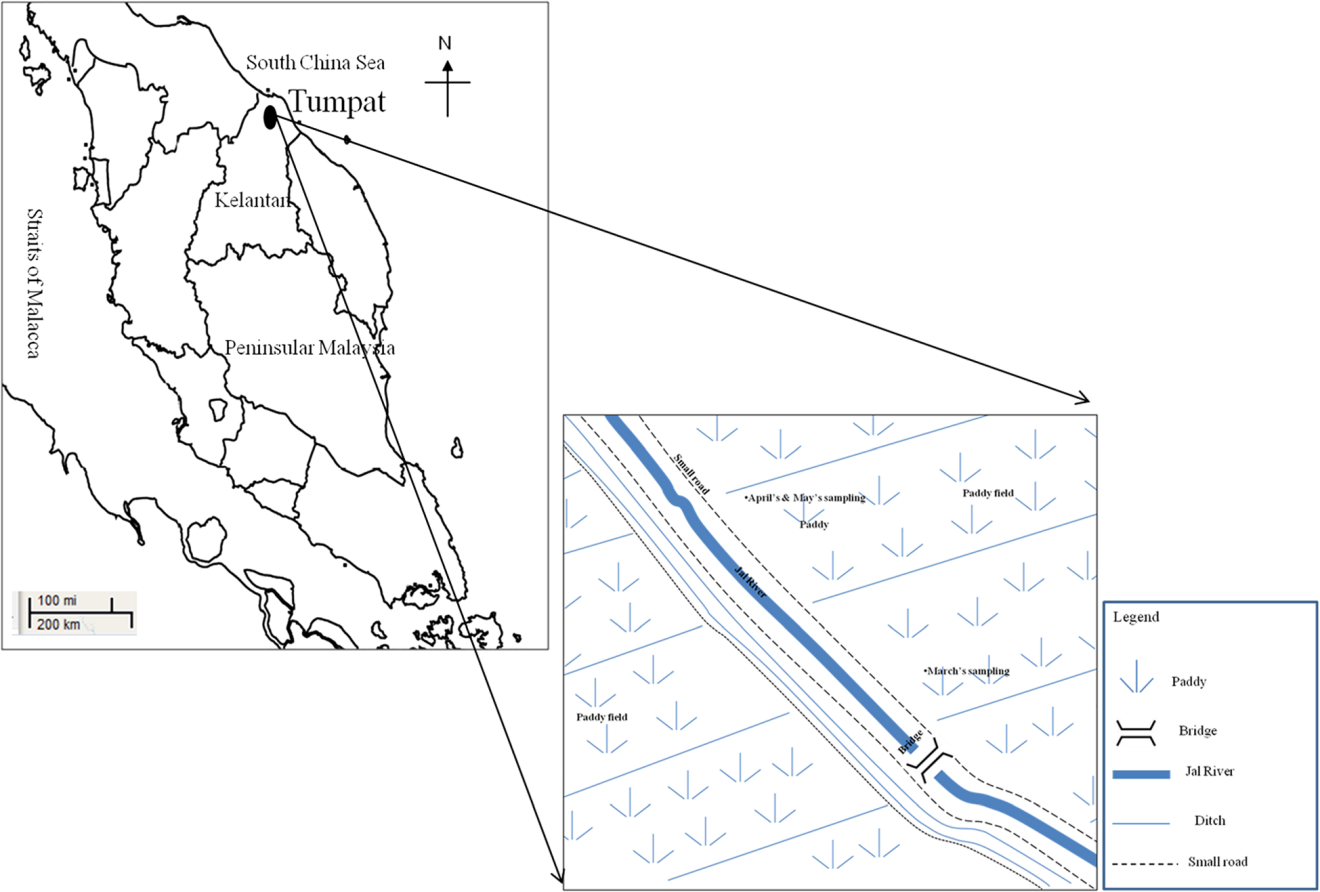
Sampling location of *M. albus* in paddy fields around Tumpat, Kelantan, Malaysia

This study selected a paddy field located at Tumpat, Kelantan as the sampling area (see Fig. 1), where paddy cultivation has been carried out for a long time, along with several vegetables planted within the perimeter. Besides, only a handful of residents had been noted there with no heavy industries. Most paddy fields in Kelantan, including the study site, are managed by the Kemubu Agricultural Development Authority (KADA) and the Jal River flows near the study area to supply water during water shortage at dry season.

### Sample collection

Before the sampling of eels, the permission for collecting the eels was acquired from the Kemubu Agricultural Development Authority (KADA) in Tumpat and from the owner of the paddy fields. In order to capture the eels, a tool called *Tukil* was used, which resembles a 36 × 2 in. semi-closed cylindrical tube comprised of a PVC pipe with a spiny entrance on one end and sealed at the opposite end. The eels were captured in year 2013 during each season; ploughing, seedling, growing, and harvesting. In capturing the eels, the *tukil* was placed at the paddy field for a whole day with some bait in it. If trapped, the eels were collected in the morning, placed in polyethylene plastic bags, sent to the laboratory, and kept in a freezer at − 20 °C until further analysis.

### Analysis of heavy metals (Zn, cu, cd, Ni, and Pb)

A study determined the concentrations of Zn, Cu, Cd, Ni, and Pb in muscle, liver, and gill of Asian swamp eel through the use of an atomic absorption spectrophotometer, coupled with flame. In fact, the validity of the outcomes was verified by the National Research Council Canada (NRCC) by issuing certification of DORM-3 and PACS-2; reference materials for fish species. Table 1 tabulates the results of the analyses that displayed exceptional recovery percentage for each metal.

**Table 1:** Measured result (μg/g dry weight ± SD) of the Certified Reference Material (CRM) for fish with its certified value for Zn, Cu, Cd, Ni and Pb

Metal	Zn^a^	Cu^a^	Cd^b^	Ni^b^	Pb^b^
Measured	46.744 ± 4.74	16.922 ± 2.02	2.676 ± 0.33	32.867 ± 5.46	171.57 ± 15.48
Certified (CRM)	51.3 ± 3.1	15.5 ± 0.63	2.11 ± 0.15	39.5 ± 2.3	183 ± 8
Recovery	91.12%	109.17%	126.82%	83.21%	93.75%

Remark: a: Certified Reference Material (DORM-3), b: Certified Reference Material (PACS-2); *SD* Standard deviation

### Determination of Metallothionein

As for analysis of MT, distilled water (DW) was used to rinse the eels a few times in order to discard slime on the skin. Next, the body sizes were recorded, which resulted as follows: the sample weights ranged between 29.0 and 271.0 g, while the total length had been in the range of 28.70 until 63.0 cm. After that, tissues from muscle, liver, and gill were extracted to determine the level of MT across the four paddy seasons. The samples were stored in a freezer at − 80 °C until further analyses. In total, fifty Asian swamp eels were examined in this study. MT was analysed in three types of tissues by adhering to the steps outlined by Viarengo et al. [17], as depicted by Amira et al. [18]. About 1 g of each tissue was homogenized in a buffer solution that consisted of 0.5 M sucrose, 20 mM Tris-HCl (pH 8.6), 0.006 mM leupeptin, 0.5 mM PMSF (phenylmethylsuphonylfuride), and 0.01% ß-mercaptoethanol. Next, centrifugation was carried out upon the samples at 30,000 *g* for 20 min at 0 °C.

### Quantification of MT by spectrophotometer

The MT in cytosol was purified via precipitation using ethanol-chloroform to discard proteins sensitive to the solvent fractionation, as well as to obtain the MT concentration by adhering to Kimura et al. [19] and Dieter et al. [20]. Next, 1 ml of the resultant supernatant was purified using 80 μl chloroform and 1.05 ml of cold ethanol (− 20 °C). After that, these samples had been centrifuged at 6000 *g* for 10 min at 4 °C. Then, 6 ml of cold ethanol and 40 μl of 37% concentrated HCl had been added, while the protein was denatured at − 20 °C for an hour. Later, the mixtures had been centrifuged again at 6000 *g* for 10 min at 4 °C. The resultant, which was in the form of pellet had been kept. Next, after discarding the supernatant, the pellet was added the following: 1 ml of buffer solution, 6 ml of cold ethanol, and 80 μl of chloroform. This mixture was also centrifuged at 6000 *g* for 10 min at 4 °C. After discarding the supernatant, the pellet was dried using N_2_ gas. Then, the pellet was suspended in a solution made of 150 μl 0.25 M NaCl, 150 μl 1 N HCl, and 4 mM EDTA. The content of MT amongst the samples was examined by using 4.2 ml of a solution that consisted of 2 M NaCl and 0.43 mM DTNB (5,5′-Dithio-Bis(2-nitrobenzoic acid)) with pH adjusted to 8 using 0.2 M Na-phosphate (NaH_2_PO_4_) at ambient temperature. Again, the mixture was centrifuged for 5 min at 3000 *g* and then, measured at 412 nm using a UV-Visible Recording Spectrophotometer Shimadzu UV-160 A Model. The concentration of MT had been determined by using glutathione (GSH), which served as reference standard, as well as calibration curve [17].

### Statistical analysis

The findings are reported in mean ± standard deviation (SD) values. The Pearson Correlation was performed to determine the concentration of MT with Zn, Cu, Cd, Ni, and Pb in the sample muscle, liver, and gill of *M.albus*. In addition, a Post-Hoc HSD Tukey test had been carried out to identify the significantly differing values upon obtaining a significant ANOVA value. Further statistical analyses had been conducted with SPSS 21.0 with *P* < 0.05 and *P* < 0.01 as the significant levels.

## Results

### Zn, cu, cd, Ni and Pb (μg/g wet weight) accumulated in liver, gills, and muscle

The findings recorded regarding levels of Zn, Cu, Cd, Ni, and Pb found in Asian swamp eels are presented in Table 2. It was revealed that Zn concentrations ranged as follows: 15.82–20.24 μg/g wet weight in liver, 24.0–29.02 μg/g in gills, and 9.14–12.09 μg/g in muscle of *M.albus*. Besides, the levels of Zn seemed higher in both liver and muscle across the four seasons. Nevertheless, an insignificant variance was noted for Zn (*p* > 0.05) in the gills (see Table 2). Meanwhile, the Cu concentrations were: 0.53–2.45 μg/g in liver, 0.09–0.17 μg/g in gills, and an average of 0.04 μg/g in muscle across the four seasons. Both muscle and gills displayed insignificant variances at *p* > 0.05 throughout the seasons (see Table 2). However, Cu appeared higher in liver for seedling season perhaps due to excessive use of fertilisers to ascertain high paddy productivity. The ranges of Cd concentrations found in the samples of eels are as follows: 0.10–0.59 μg/g in liver, 0.09–0.66 μg/g in gills, and 0.08–0.16 μg/g in muscle. In fact, Table 2 presents that the Cd levels had been the lowest in samples, as compared to other metals in the eel tissues. Next, the levels of Ni in the tested tissues across the seasons are as recorded: 1.75 μg/g in liver, 9.21–15.92 μg/g in gills, and 1.40–2.57 μg/g in muscle (see Table 2). Besides, the Ni showed insignificant variance for gill tissues at *p* > 0.05. Lastly, Pb portrayed insignificants outcomes for all samples across all seasons (see Table 2).

**Table 2:** Zn,Cu,Cd,Ni and Pb concentration in livers, gills and muscle of Asian swamp eels on 4 different paddy seasons

Metal	Tissue	Plowing	Seedling	Growing	Harvesting
Zn	Liver	20.24 ± 2.27^a^	19.65 ± 1.87^ab^	17.30 ± 1.65^bc^	15.82 ± 0.92^c^
	Gills	29.02 ± 6.51^a^	27.92 ± 2.10^a^	24.00 ± 3.42^a^	25.46 ± 4.28^a^
	Muscle	11.47 ± 2.51^ab^	12.09 ± 1.09^a^	11.69 ± 1.19^ab^	9.14 ± 0.61^b^
Cu	Liver	1.75 ± 1.20^ab^	2.45 ± 0.78^a^	1.00 ± 0.85^c^	0.53 ± 0.31^c^
	Gills	0.17 ± 0.09^a^	0.09 ± 0.01^a^	0.13 ± 0.11^a^	0.09 ± 0.07^a^
	Muscle	0.04 ± 0.01^a^	0.04 ± 0.01^a^	0.04 ± 0.01^a^	0.04 ± 0.01^a^
Cd	Liver	0.10 ± 0.08^bc^	NA	0.59 ± 0.25^a^	0.37 ± 0.11^ab^
	Gills	NA	NA	0.66 ± 0.60^a^	0.09 ± 0.07^b^
	Muscle	NA	NA	0.16 ± 0.08^a^	0.08 ± 0.02^b^
Ni	Liver	3.71 ± 1.63^a^	2.09 ± 0.46^ab^	1.75 ± 0.76^b^	2.42 ± 0.65^ab^
	Gills	15.92 ± 8.35^a^	9.21 ± 2.74^a^	7.41 ± 2.83^a^	7.47 ± 1.53^a^
	Muscle	2.57 ± 0.60^a^	1.94 ± 0.79^ab^	1.4 ± 0.46^b^	2.14 ± 0.35^a^
Pb	Liver	0.47 ± 0.17^a^	0.35 ± 0.01^a^	0.54 ± 0.32^a^	0.36 ± 0.04^a^
	Gills	1.53 ± 0.84^a^	0.71 ± 0.20^a^	1.14 ± 0.95^a^	0.58 ± 0.22^a^
	Muscle	0.33 ± 0.05^a^	0.34 ± 0.01^a^	0.34 ± 0.10^a^	0.35 ± 0.01^a^

Remark:Values are given in mean ± S.D

*Post-Hoc*: Mean metal concentrations of different parts of tissues sharing a common letter

for a particular metal present no significant differences, *p > 0.05*

### Metallothionein concentration in liver, gills and muscle

Table 3 shows the levels of MT recorded for all samples of eels in the form of mean ± SD and expressed in μg/g MT. As a result, the levels of MT content refer to the following order: liver > gills > muscle. Nonetheless, liver seemed to disclose higher levels of MT, when compared to muscle and gills, except for the growing season. Meanwhile, an insignificant variance was discovered for MT in gill tissues at *p* > 0.05, but significant variances were revealed for MT in muscle and gills across all seasons at *p* < 0.05.

**Table 3:** Mean concentration of MT (μg/g ± S.D) in the livers, gills, and muscle of Asian swamp eels based on four paddy seasons

[MT]			
Season	Livers	Gills	Muscle
Plowing	2278.38 ± 875.65^*a*^	1535.5 ± 408.38^a^	1593.5 ± 387.14^ab^
Seedling	1661.34 ± 393.58^*b*^	1525.37 ± 415.6^a^	1504.12 ± 528.11^ab^
Growing	1707.23 ± 508.22^b^	1776.38 ± 398.69^a^	1339.95 ± 232.33^b^
Harvesting	1900.81 ± 450.27^ab^	1671.15 ± 249.8^a^	1681.52 ± 214.32^a^

Remark: Objects indicated with different alphabet *p* < 0.05, objects indicated with same alphabet *p* > 0.05

### Metallothionein concentration with Zn, cu, cd, Ni and Pb in liver, gills and muscle

The results from Pearson correlation displayed significantly positive results (*p* < 0.05) for MT-Zn, MT-Cd, and MT-Ni in the liver samples, but exceptional for MT-Cu and MT-Pb. The muscle and gill tissues had correlations that were moderate and insignificant variances at *p* > 0.05 for MT-Zn, MT-Cu, MT-Cd, MT-Ni, and MT-Pb (see Table 4).

**Table 4:** Pearson’s Correlation between MT with Zn, Cu, Cd, Ni and Pb in three different tissues of Asian swamp eels

	Livers	Gills	Muscle
MT-Zn	0.77*	−0.09	0.08
MT-Cu	0.30	0.10	0.25
MT-Cd	0.49*	0.28	0.10
MT-Ni	0.55*	0.01	0.20
MT-Pb	0.006	0.12	−0.08

Remark: *Correlation significant at *p* < 0.05

## Discussions

This study found that the Asian swamp eels had the lowest metal concentrations for muscle tissues. For example, Mulk et al. [21] revealed the lowest heavy metals accumulation in fish muscle tissues that varied by types of fish [22]. Meanwhile, Cu and Zn were found in liver, whereas non-significant metals (Pb and Ni) were highly concentrated in gill tissues across the paddy seasons. As for Cd, its levels varied across the seasons with fluctuating recordings for all tissue samples.

Zhao et al. [23] reported that on the existence of metals discovered in liver is highly linked to metabolism functions. As for this current research, Cu and Zn were high in liver tissue due to MT; a binding protein [24] that stores metals so as to cater to the needs of enzymes and metabolic requirements [15, 25]. Zn was high in liver tissues across the four seasons due to essential biological needs [26] for fish samples taken from paddy fields in Tumpat [27]. Similarly, Zn was found in high concentration in gill tissues across the four seasons, mainly because this organ is the initial target of metals in water [28]. According to Reid and Mcdonald [29], the negatively-charged surface of the gills offers a viable interaction platform between metal and gill, especially for positively-charged metals. As for this study, the high concentrations of Zn discovered in gill and liver tissues signify that the Zn pathway has the dietary source, thus the spread to liver from gill. It is also important to highlight that paddy fields are inhabited by many other smaller freshwater invertebrates, which eases eels to seek for food. On top of that, the samples had Pb and Ni in gill tissues. This is in line with that discovered by Kargin [30] for *Capoeta barroisi*. The Pb and Ni bioaccumulation may have taken place in the gills due to the contaminated water at the study area [31]. In fact, Pb is a metal that is commonly found in the environment [32]. As for Cd, its levels appeared lower in gill tissue, as compared to that reported by El-Moselhy et al. [33]. The vast gill surface area promotes toxic metals diffusion in a rapid manner [34]. Hence, it has been hypothesised that the metals found in gill usually originate from water [33]. The metals in the gills, after that, are either transferred to other body parts, especially liver for detoxification or discarded into water [35].

Meanwhile, Velusamy et al. [36] asserted that the variance in uptake of elements among species depends on several fish biological traits, for instance, mobility, habitat, and trophic aspects. Hence, as Asian swamp eels live in paddy fields with varied seasons, their exposure to heavy metals is indeed high. Therefore, these eels project high levels of metal in their tissues samples. Furthermore, as these eels have increased longevity, these metals accumulate in their bodies as pollutants.

Apart from metabolic status, growth rate, ecological needs, and feeding habits; another factor that influences their exposure to metals is their patterns in life history [37]. Moreover, Suresh et al. [38] found that trace metals are seldom noted with uniform dispersion in the fish tissues, but accumulate in certain organs. This is in line with that reported by Hazrat and Ezzat [22] that vast factors dictate the accumulation of metalloids and heavy metals in freshwater aquatics, including fish traits and other external factors in their surroundings.

At recent times, the application of MT as a biomarker has begun to increase to evaluate biological impacts due to metals exposure [39]. As such, MT could be detected in several parts of the fish, such as intestine, liver, and gills [40]. Table 3 shows that MT is abundantly present in gill, muscle, and liver tissues. As for this research, the levels of MT are higher in liver, as compared to muscle and gill tissues, due to the role of liver in storing metals for detoxification purposes [41]. Similarly, Naji et al. [42] reported higher MT content in liver of Mozambique tilapia, than that in gills. Thus, it is a common scenario to detect high levels of MT in eel liver due to exposure to metals (Cd, Cu, and Zn) [43]. Nevertheless, compared to several other studies pertaining to discovery of MT in *M.albus*, this study displayed only slight variances for MT levels between gill and liver tissues. This could be due to the suitability of examining gills within the context of monitoring the environment [44]. Moreover, one vital outcome from this study is that although Cu, Cd, Ni, and Pb were detected in the tissues analysed, they did not necessarily display correlation with the level of MT in muscle and gill tissues. This indicates that the levels of MT depend on the type of tissue [31]. Amérand et al. [45] reported that high levels of MT found in muscle tissues could reflect the occurrence of intense oxidising metabolism activities that release free radicals into the system, thus inducing the synthesis of MT [46–48].

Furthermore, the positive relationship between Zn and MT in liver signifies the function of MT in homeostasis of Zn and the fact that Zn is a constituent element of MT [49]. In studying perch, *Perca fluviatilis*, Hogstrand et al. [50] revealed a positive relationship between Zn and MT (*r* = 0.75, *P* < 0.001), and a significantly lower and positive link between Cu and MT in the liver tissues, which was in line with the low Cu accumulation in liver (see Table 3). Hence, MT binds well with toxic metal, particularly Zn, but not as well as with Cu. Besides, the association of MT levels and Cd for this study in liver tissues is attributable to dietary uptake along the gastrointestinal tract of the Asian swamp eel. Additionally, McGeer et al. [51] asserted that Cd ions pass through intestinal walls with the aid of metal carriers, for instance, Ca, Cu, Zn, and Fe channels. Upon reaching the blood stream, Cd ions are carried by proteins - MT [52]. As liver seems to be the initial organ the metals are sent to, escalated accumulation of Cd is noted in liver, when compared to other tissue types. Next, MT and Ni also displayed a significantly positive relationship in liver since the MT refers to metal biomarker [53]. Cosson [54] mentioned that metal binding on MT platform does not only rely on the number of cells. As for Pb and MT, insignificant correlation was noted in the liver, perhaps due to either exceeding binding capacity of MT or incorporation of non-MT proteins with low molecular weight for sequestration of metal [4]. Meanwhile, the absence of link between metals and MT in muscle and gill tissues for this study is attributable to the presence of metal-protein complexes, instead of free ions [55, 56], or due to the low quantity of free ions to promote synthesis of MT.

## Conclusions

As a conclusion, the outcomes of this study highlight the following: (1) the high presence of Zn was noted in gill and liver tissues, while Cu solely in liver; (2) accumulation of non-essential metal, such as Cd, had been low in all tissues, while Pb and Ni were abundant in gill tissues across all seasons; (3) the level of MT was higher in liver, when compared to muscle and gill tissues throughout the four paddy seasons; (4) MT-Zn, MT-Cd, and MT-Ni exhibited high correlations (*p* < 0.001), while significantly low correlation for MT-Cu in liver can be associated to the high metal-binding capacity exerted by MT with Zn, Cu, Cd, and Ni, hence emphasising the role of MT as a biomarker for exposure of metal in this particular eel species.

## Abbreviations

%: Percentage
°C: Degree celcius
AAS: Atomic Absorption Spectrophotometer AAS
Cd: Cadmium
Cm: Centimetre
CRM: Certified reference material
Cu: Copper
DTNB: 5,5-dithiobis-2-nitrobenzoic acid
DW: Distilled water
G: Gram
M: Molarity
ml: Millilitre
mM: Milimolar
MT: Metallothionein
Ni: Nickel
Pb: Lead
PMSF: Phenylmethylsuphonylfuride
SD: Standard deviation
Zn: Zinc
μg/g: Microgram per gram
μl: Microlitre
